# Mycophenolate-Induced Colitis: A Rare Side Effect

**DOI:** 10.7759/cureus.18250

**Published:** 2021-09-24

**Authors:** Baha Aldeen Bani Fawwaz, Ahmad Aldwairy, Aimen Farooq

**Affiliations:** 1 Internal Medicine, AdventHealth Orlando, Orlando, USA; 2 Internal Medicine, MedStar Washington Hospital Center, Washington, USA

**Keywords:** renal transplant recipient, mycophenolate side effects, terminal ileitis, mycophenolate, colitis

## Abstract

Mycophenolic acid (MPA) is a well-known immunosuppressive medication that is widely used in solid organ transplant recipients. This medication is known to have various gastrointestinal (GI) side effects. Some of those side effects are simple and temporary like nausea; on the other hand, it can also lead to more serious side effects like colitis. We herein report a case of MPA-induced colitis that presented with severe diarrhea. Unexpectedly, the endoscopic appearance of the colon was normal. Moreover, pathology findings were nonspecific. Symptoms had resolved after discontinuation of the medication. MPA-induced colitis is a rare side effect with no established guidelines for management or treatment. Furthermore, the nonspecific endoscopic and microscopic findings make it a diagnostic dilemma.

## Introduction

Mycophenolic acid (MPA) is a well-known immunosuppressive agent for its use in post-organ transplants and various autoimmune disorders. Immunosuppressive properties of MPA arise mainly from its inhibitory effects on the rate-limiting enzyme for purines synthesis in lymphocytes [1]. However, other cells in the body, including gastrointestinal (GI) epithelium, share the same pathway for purine synthesis, making it also vulnerable to MPA effects. MPA can lead to cytotoxic and cytostatic effects on the GI epithelium, impairing fluid absorption resulting in diarrhea [2]. The incidence of diarrhea with MPA has been reported to be as high as 53%. However, MPA-induced colitis and terminal ileitis are rare, <1% [3]. We present a case of microscopic colitis and terminal ileitis that is induced by MPA used in a kidney transplant recipient.

## Case presentation

A 53-year-old female patient with a medical history of polycystic kidney disease status post renal transplant (March 2019), on MPA and Belatacept, and a recent shingles infection presented to the emergency department (ED) complaining of diarrhea for three weeks. She has been having 5-6 episodes of loose stools every day with occasional streaks of blood associated with non-bloody vomiting at least once a day. The patient reported significant decrease in her oral intake resulting in 10 pounds weight loss over that period. The patient denied abdominal pain, fevers, chills, and urinary symptoms. It is of note that four weeks prior to admission, the patient had noted a rash over the right buttock and vaginal area for which she was prescribed topical medications by her primary care provider. Vaginal rash improved but buttock rash continued to worsen and became painful. The patient went to the ED where she was diagnosed with shingles and superimposed bacterial infection. The patient was started on cephalexin and the rash subsequently improved.

Upon presentation, the patient was afebrile with a heart rate of 98 bpm and blood pressure of 82/47 mmHg. Physical examination was significant for an ill-appearing woman with dry mucous membranes and crusted lesions over right T6-T7 and bilateral S1-S2 dermatomes. The abdomen was non-tender with no blood noticed on the digital rectal exam. A vast array of blood tests were done at admission and during initial workup and are detailed in Table 1. The patient's baseline creatinine is 2.1 mg/dL.

**Table 1:** Laboratory investigations. CMV, Cytomegalovirus; PCR, polymerase chain reaction; Ag, antigen; EIA, enzyme immunoassays

Tests	Patient value	Normal value
Hemoglobin	10.5 g/dL	11.4-14.7 g/dL
White blood count	10.26 10*3/µL	4.40-10.50 10*3/µL
Bicarbonate	9 mmol/L	24-32 mmol/L
Creatinine	3.35 mg/dL	0.60-1.20 mg/dL
Glomerular filtration rate	15 mL/min/{1.73_m^2^}	>60 mL/min/{1.73_m^2^}
Lipase	162 units/L	10-60 units/L
Liver function tests	Normal	
CMV PCR	Negative	
BK virus PCR	Negative	
Fecal bacterial and viral panel	Negative	
Fecal ova and parasites	Negative	
Clostridioides difficile Ag	Negative	
Clostridioides difficile toxin EIA	Negative	

**Figure 1:**
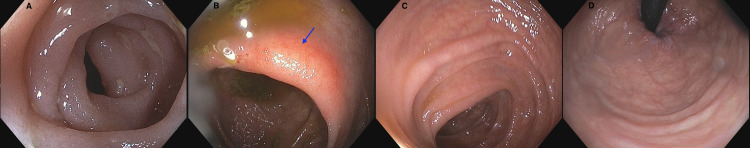
Colonoscopy findings of the patient. Endoscopic appearance of terminal ileum (A), Ileocecal valve (B), descending colon (C), and rectum (D).

CT of the abdomen and pelvis without contrast showed liquid stool in the colon but no evidence of colitis. On the third day of admission, the MPA dose was decreased (1,080 mg/day to 720 mg/day). Forty-eight hours after lowering the MPA dose, the patient reported significant improvement in her symptoms. The patient underwent a colonoscopy on day 5 which showed ileitis, mild inflammation at the ileocecal valve, and the entire examined colon was normal (Figures 1A-1D). Biopsies from the ileum and colon were sent for pathology.

**Figure 2:**
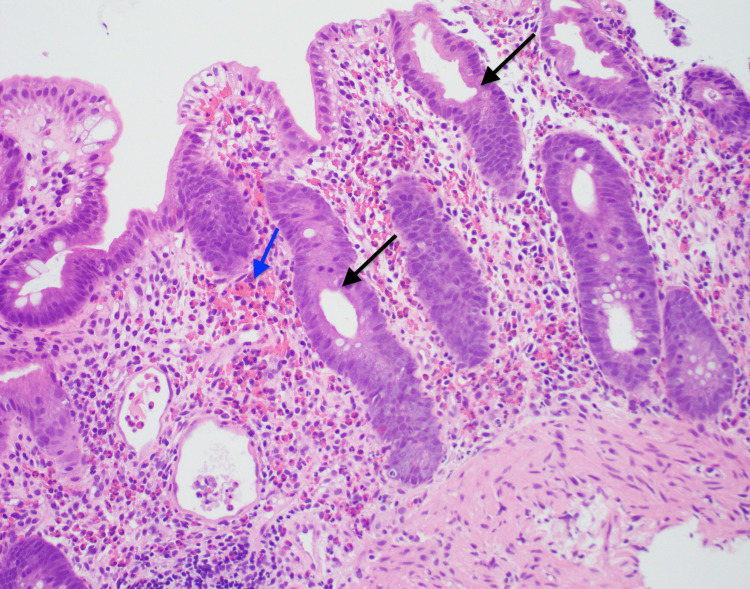
Pathology findings Hematoxylin and eosin-stained sections of colonic mucosa showing dilated crypts (black arrows) with edema and numerous eosinophils (blue arrow) in lamina propria. Rare pigmented macrophages are also present.

Pathology revealed active ileitis with focal ulceration in terminal ileum biopsy, active colitis, and cryptitis with mild architectural distortion in the ileocecal valve as well as transverse, right and left colon biopsies (Figure 2). The patient was started on Azathioprine and MPA was discontinued. She was discharged on day 7 after a complete resolution of diarrhea.

## Discussion

MPA is known to have various GI side effects that range from abdominal pain and nausea to more profound adverse effects like pancreatitis, colitis, or peritonitis [3]. A common practice in renal transplant recipient patients is to stop MPA when patients experience severe diarrhea. The dose-dependent effect of MPA on GI tract is not well established. Our case puts a spotlight on possible dose-dependent effects of MPA and that lowering the dose might be sufficient to limit some of the side effects. However, maintaining therapeutic levels of MPA should also be taken into consideration. 

Most of MPA GI’s side effects occur in the first six months after the initiation of the therapy [4]. In our case, the onset of the diarrhea more than 12 months after initiation of MPA raises the question if this new-onset colitis might be provoked by supratherapeutic levels of MPA secondary to acute kidney injury, since the majority of MPA is eliminated in the urine in the form of MPA-glucuronide (MPAG) (inactive metabolite, >60%) and MPA unchanged (3%) [5]. Dehydration from diarrhea might also have initiated a vicious cycle of dehydration, decreased renal perfusion, and MPA clearance leading to further increase in MPA levels. Moreover, the incidence of MPA-induced colitis is higher in renal transplant recipients compared to other solid organ transplant recipients, as kidney function is usually lower in the postoperative period [4]. However, Mycophenolate dosage in the former is usually higher and the relative scarcity of literature on the latter can also explain this. Currently, there are no recommended dosage adjustments for MPA in patients with renal impairment. Nonetheless, it is recommended to monitor patients carefully for concentration-dependent adverse effects in this population of patients [3]. In our case, the dosage was reduced by 33% and symptoms started to improve 48 hours after dosage reduction.

One of the limitations in MPA colitis is the variance and the non-specificity of the macroscopic and histological findings, which makes it a diagnostic dilemma. For instance, in our case the pathology findings (Figure 2) were not suggestive of ischemic bowel disease or inflammatory bowel disease but drug-induced etiology could show similar findings. Also, there are no specific guidelines for the treatment or management of suspected MPA-induced enterocolitis. Discontinue-and-see approach versus lower the dose-and-see approach has been described in literature. Infliximab and/or prednisone have been also used for refractory cases [2,6,7]. An approach to severe diarrhea in renal transplant patients has been described in DIDACT study results [8]. This approach suggests alterations and adaptations of immunosuppressive therapy and ruling out infectious etiologies before proceeding with colonoscopy. Colonoscopy and mucosal biopsies would be appropriate for persistent diarrhea [8]. This approach would circumvent patients from getting invasive procedures that could have been avoided by medications adjustment. Not to mention lowering the financial burden on patients as well as preserving resources.

## Conclusions

MPA is one of the most commonly used immunosuppressant medications in renal transplant recipients. Although this medication has been well known for its GI side effects, no guidelines have been developed to aid in approaching or managing such events. Discontinuing any possible offending agents in patients with suspected medication-induced etiology would be a reasonable approach. Notwithstanding that patients with persistent or severe symptoms might be investigated more invasively simultaneously with discontinuing such agents. Infliximab and prednisone remain the main therapeutic options in refractory confirmed MPA-induced colitis cases.
